# Mitochondrial Dynamics and Microglia as New Targets in Metabolism Regulation

**DOI:** 10.3390/ijms21103450

**Published:** 2020-05-13

**Authors:** Martina Chiurazzi, Martina Di Maro, Mauro Cozzolino, Antonio Colantuoni

**Affiliations:** 1 Program in Integrative Cell Signaling and Neurobiology of Metabolism, Yale University School of Medicine, New Haven, CT 06520, USA; 2 Department of Cellular and Molecular Physiology, Yale University School of Medicine, New Haven, CT 06520, USA; 3 Department of Clinical Medicine and Surgery, University of Naples “Federico II”, 80131 Naples, Italy; martinadimaro@hotmail.it (M.D.M.); colantuo@unina.it (A.C.); 4 Department of Obstetrics, Gynecology and Reproductive Sciences, Yale School of Medicine, New Haven, CT 06520, USA; mauro.cozzolino@yale.edu; 5 Department of Obstetrics and Gynecology, Rey Juan Carlos University, Calle Tulipán, Móstoles, 28933 Madrid, Spain; 6 IVIRMA, IVI Foundation, Health Research Institute La Fe, Avenida Fernando Abril Martorell, 106, 46026 Valencia, Spain

**Keywords:** energy homeostasis, hypothalamus, mitochondrial dynamics, microglia, obesity

## Abstract

Energy homeostasis regulation is essential for the maintenance of life. Neuronal hypothalamic populations are involved in the regulation of energy balance. In order play this role, they require energy: mitochondria, indeed, have a key role in ensuring a constant energy supply to neurons. Mitochondria are cellular organelles that are involved in dynamic processes; their dysfunction has been associated with many diseases, such as obesity and type 2 diabetes, indicating their importance in cellular metabolism and bioenergetics. Food intake excess can induce mitochondrial dysfunction with consequent production of reactive oxygen species (ROS) and oxidative stress. Several studies have shown the involvement of mitochondrial dynamics in the modulation of releasing agouti-related protein (AgRP) and proopiomelanocortin (POMC) neuronal activity, although the mechanisms are still unclear. However, recent studies have shown that changes in mitochondrial metabolism, such as in inflammation, can contribute also to the activation of the microglial system in several diseases, especially degenerative diseases. This review is aimed to summarize the link between mitochondrial dynamics and hypothalamic neurons in the regulation of glucose and energy homeostasis. Furthermore, we focus on the importance of microglia activation in the pathogenesis of many diseases, such as obesity, and on the relationship with mitochondrial dynamics, although this process is still largely unknown.

## 1. Introduction

Excess weight and obesity, as well as hypertension, smoking, diabetes, and a sedentary lifestyle are among the most important risk factors for several diseases, especially cardiovascular diseases and cancer, which are, currently, the leading causes of morbidity and premature mortality in the world [1,2]. Currently, positive results in mortality reduction were obtained through hypertension, hypercholesterolemia, and smoking prevention and treatment [3]. Exceptions to these positive trends are represented by obesity and diabetes, which, on the other hand, are constantly growing [4]. Many studies have shown that excessive calorie intake, reduced consumption of high-fiber foods, and a sedentary lifestyle are among the main risk factors causing obesity. Furthermore, some genes involved in the pathogenesis of obesity such as the melanocortin-4 receptor gene, responsible for the most frequent form of monogenic obesity induced by a single gene mutation, have been identified [5]. In particular, in a study on Dutch children, it was observed that about 2% of obese children showed a mutation in the MC4R gene in homozygosity and heterozygosity, presenting a phenotype characterized by being extremely overweight and hyperphagia. Children with melanocortin-4 receptor (MC4R) heterozygous mutations, however, were difficult to distinguish from obese children without mutations [6]. Numerous scientific data have also shown that specific alterations in the composition and function of the intestinal microbiome in humans can be linked to the pathogenesis of metabolic disorders. Furthermore, considering that the composition of the microbiota can be influenced by the diet, the manipulation of the microbiota is thought to represent a therapeutic strategy for the treatment and prevention of obesity [7]. Obesity induced by an unbalanced diet, characterized by an excess of fats and carbohydrates, puts the individual at risk of an inflammatory state and in a condition of oxidative stress. Inflammation is implicated in the pathophysiology of many of the complications associated with obesity, such as cardiovascular risk, metabolic syndrome, insulin resistance, and diabetes mellitus [8,9].

The modulation of appetite and satiety, as well as the modulation of energy expenditure and the accumulation of energy in the adipose tissue represent some of the homeostatic processes involved in the regulation of body weight. In this complex homeostatic system, there are some signals that transmit information to the central nervous system (CNS), contributing to the long- and short-term regulation of body weight [10].

The regulation of the homeostasis of systemic energy is an essential function exerted also by many hormones derived from adipocytes. Adiponectin is an endocrine factor synthesized and released from adipose tissue that plays an important role in improving insulin sensitivity in the liver and skeletal muscles and in regulating energy expenditure, suggesting an important role of this factor in the prevention of obesity and related diseases such as insulin resistance/type 2 diabetes and cardiovascular disease. Its effects are mediated by two adiponectin receptor isoforms, AdipoR1 and AdipoR2 [11,12].

Recent studies, indeed, have demonstrated the important role of the CNS in regulating energy and glucose metabolism in response to changes in peripheral circulating signals, such as signals of a hormonal nature (e.g., leptin and insulin), as well as nutrients (glucose and fatty acids). The central nervous system, glial cells, and hypothalamus play a fundamental role in controlling metabolism [13,14,15,16]. The mitochondrial function plays an important role in this regulation. Despite the mechanisms not yet being fully understood, mitochondrial dysfunction in hypothalamic neurons seems to be able to contribute to the development of many metabolic diseases, such as obesity and type 2 diabetes [17,18,19]. Furthermore, changes in mitochondrial metabolism, as observed in inflammation, have been shown to contribute also to the activation of the microglial system in numerous diseases; this system appears, indeed, to be important in metabolic control [20,21,22]. In this review, we summarize the link between mitochondrial dynamics and hypothalamic neurons in the regulation of glucose and energy homeostasis. Furthermore, we focus on the importance of microglia activation in the pathogenesis of many diseases, such as obesity and insulin resistance, and on the relationship with mitochondrial dynamics, although this process is still widely unknown.

## 2. Obesity and Hypothalamic Inflammation

Obesity is a major risk factor for associated comorbidities, such as cardiovascular diseases, type 2 diabetes mellitus, cancer, and mortality. Currently, obesity has reached global epidemic proportions in both adults and children, resulting in a substantial financial burden on healthcare. The World Health Organization (WHO) defines obesity as an excessive accumulation of fat diagnosed with a body mass index (BMI) ≥ 30 kg / m^2^, which could compromise health [23,24,25].

Moreover, obesity is defined as a condition of chronic inflammation and presents a multifactorial etiology, such as genetic factors and hormone imbalance, as well as diet and the environment [26].

Inflammation is a protective response of the body to maintain the homeostasis of tissues and organs and can be of two types: short-lived acute inflammation, characterized by edema and migration of leukocytes, or long-lasting chronic inflammation, characterized by the presence of lymphocytes and macrophages and by the proliferation of blood vessels and connective tissue.

Chronic inflammation induces the secretion of inflammatory adipokines from adipose tissue, such as interleukin (IL-6), tumor necrosis factor-α (TNF-α), chemoactive protein monocyte-1 (MCP- 1), and resistin [8,27].

The consumption of an unbalanced diet, characterized by an excess of fats and carbohydrates, can cause inflammation in the peripheral organs, in particular in the adipose tissues [8,9,28]. The consequences of dysfunctional adipose tissue closely linked to obesity and metabolic disorders are inadequate angiogenesis, hypoxia, inflammation, as well as fibrosis. In a pathological obesity condition, adipocyte hypertrophy and hypoxia are able to cause inflammation, inducing the production of many cytokines and chemokines, involved in the beginning and development of the inflammatory response associated with obesity in adipose tissue and obesity-induced insulin resistance [29,30]. Furthermore, this condition induces a reduction of the production of adiponectin, important for its anti-inflammatory function and for its ability to improve sensitivity to insulin and regulate energy expenditure, predisposing to a pro-inflammatory state and oxidative stress, thus contributing to the pathogenesis of obesity [8,31]. Many studies have shown that obesity induced by a high-fat diet (HFD) causes chronic hypothalamic inflammation [32,33,34].

Hypothalamus, an important coordinator of the endocrine system and the autonomic nervous system, located near the pituitary gland, has been considered, indeed, a key area of brain for the regulation of metabolism, due to neurons effective at perceiving, integrating, and responding to numerous metabolic signals, such as hormones, especially leptin, ghrelin, and insulin, as well as nutrients, such as glucose, amino acids, and fatty acids. Hypothalamus plays an essential role in modulating feeding behavior and energy expenditure [35,36,37,38].

The numerous hypothalamic nuclei and neuronal circuits, such as arcuate nucleus (ARC), the ventromedial (VMH), paraventricular, dorso-medial, and lateral hypothalamic areas, have been widely investigated to understand their role in the regulation of metabolism [16,39,40]. Hypothalamic neuroinflammation can cause changes in neurons of arcuate nucleus, such as those releasing agouti-related protein (AgRP) and proopiomelanocortin (POMC), which are involved in the regulation of energy homeostasis sensing and integrating numerous metabolic signals [16,17,18]. The central nervous system and its hypothalamic nuclei appear to be linked to the circadian clock that controls many genes involved in cellular metabolism, playing an important role in homeostatic regulation. Animal model studies have shown that disruption of the main clock located in the suprachiasmatic nucleus (SCN) of hypothalamus can disrupt rhythmic behaviors, such as feeding. These data suggest that changes in the circadian rhythm associated with low-grade systemic inflammation, initially observed in adipose tissues, can be related to the increase in obesity and metabolic disorders. [41,42,43].

## 3. Mitochondrial Dynamics: Fusion and Fission in Metabolic Regulation

Central nervous system cell populations are involved in the regulation of energy balance, playing an essential role in the maintenance of life. To exert this role, CNS cells require energy, so they are related to the mitochondrial system to receive a constant energy supply, even though they convey the changes in mitochondrial metabolism [16,18].

Mitochondria are important organelles for maintaining the normal physiological function of tissue cells. In the past, these organelles were considered exclusively the “powerhouse” of the cells, because they were able to produce the energy requirements for cellular metabolism by oxidative phosphorylation. Subsequently, some studies have revealed that mitochondria can be also involved in several other physiological processes, such as programmed cell death, innate immunity, autophagy, redox signaling, calcium homeostasis, and reprogramming of stem cells. Mitochondria are strongly involved in oxidative stress, and they represent the primary source of reactive oxygen species (ROS). The structure of these organelles is characterized by a double membrane system: an outer mitochondrial membrane (OMM) facing the cytosol and an inner mitochondrial membrane (IMM) facing the mitochondrial matrix containing mitochondrial DNA (mtDNA). Moreover, the two membranes (OMM and IMM) delimit the intermembrane space (IMS) [44]. These highly dynamic organelles are able to change their shape and distribution by undergoing either fission or fusion, in response to physiological or metabolic conditions. These processes are fundamental to determine the morphology and volume of the mitochondria to permit their immediate adaptation to cell energy requirements [45]. Mitochondrial fusion is a process controlled by a family of membrane-anchored proteins called mitofusin 1 (Mfn1) and mitofusin 2 (Mfn2), localized on mitochondrial outer membranes; moreover, a single inner-membrane-specific protein named optic atrophy 1 (optic atrophy 1, Opa 1-mitochondrial dynamics as GTPase) plays a role. Mfn1 and Mfn2 belong to the transmembrane GTPase family and require GTPase activity for their function. These proteins play a role at different time points in the mitochondrial fusion process. Mfn1, indeed, is important in the modulation of mitochondrial fusion; moreover, Mfn1 mediates Opa-1-driven mitochondrial fusion, showing both transcriptional and posttranscriptional involvement. Mfn2, on the other hand, protects brain against neurodegeneration in different regions, such as cerebellum, hippocampus, and cortex, as well as in different populations, especially dopaminergic neurons.

**Figure 1:**
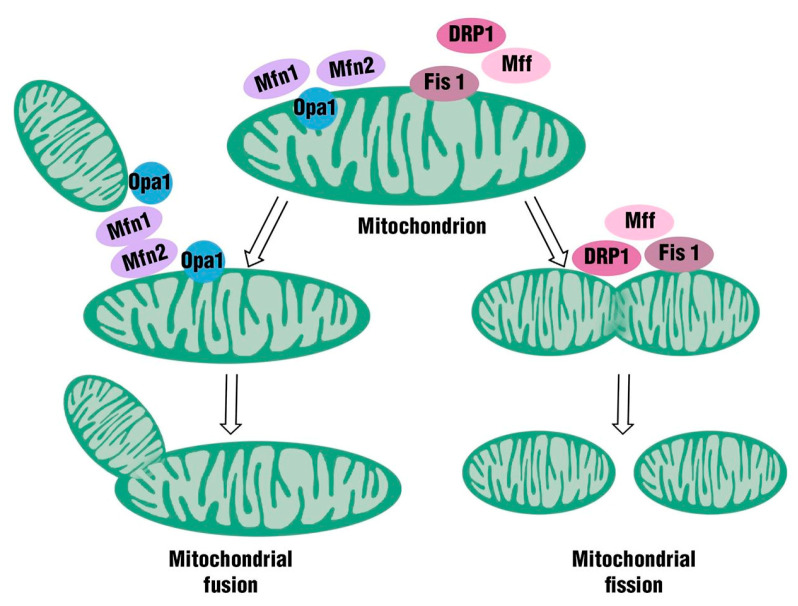
Mitochondria are important organelles for maintaining the normal physiological function of tissue cells; they are able to change their shape and distribution, undergoing either fission or fusion.

Conversely, mitochondrial fission is a process controlled by fission protein 1 (Fis1) and mitochondrial fission factor (Mff), located on the outer mitochondrial membrane, as well as by Drp1 (dynamin-related protein 1), located mainly in the cytosol. Mitochondrial dynamics are highly regulated processes [46,47] (Figure 1).

Drp1 and Fis1 are proteins involved in the regulation of mitochondrial fission. Drp1 is a cytosolic protein regulated by post-transcriptional modifications, such as phosphorylation and SUMOylation; it is recruited into the outer mitochondrial membrane where cleavage occurs. This causes hydrolysis of GTP, thus promoting its division; FIS1, on the other hand, is found mainly in the outer mitochondrial membrane and is connected via its terminal COOH to the outer mitochondrial membrane [48,49,50,51].

Mutations in some mitochondrial proteins appear to be among the main causes of some metabolic diseases, such as obesity and type 2 diabetes. In particular, a reduced expression of Mfn2 has been shown to induce impairment in mitochondrial fusion and is involved in glucose intolerance and in the increase of hepatic gluconeogenesis, confirming that this protein plays a fundamental role in insulin signaling and in glucose homeostasis. Several data, likewise, have shown that a deletion or a mutation of Opa1, with consequent impairment of the mitochondrial fusion process, can influence, in a negative manner, the insulin-stimulated mitochondrial energy metabolism, involved in the regulation of obesity and diabetes [52]. A study has shown an age-associated loss of Opa1 in muscle of sedentary subjects with consequent muscle inflammation, ER stress, and fibroblast growth factor secretion 21 (FGF21) [53]. A deletion or a mutation of dynamin-related protein 1, likewise, appears to be at the basis of the pathogenesis of insulin resistance involved in obesity and type 2 diabetes, even though further studies are required to determine the regulatory mechanisms of Drp1 [52] (Table 1).

Consequently, the development of many diseases, such as obesity and type 2 diabetes, indeed, appears to be caused by changes in mitochondrial dynamics in the organs involved in energy metabolism, such as skeletal muscle, white adipose tissue, pancreas, and liver. Moreover, mitochondria are able to induce the development of obesity, causing alterations in ATP levels and cell signaling, oxidative stress, ER stress, and inflammation, important in the onset of obesity and in maintaining energy homeostasis [54,55,56].

## 4. Mitochondrial Dynamics and Hypothalamic Neurons

**Figure 2:**
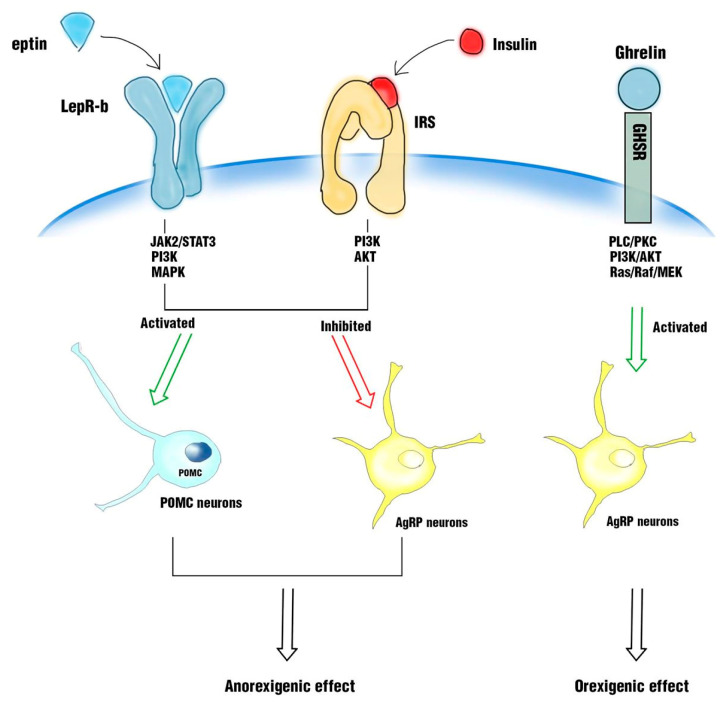
In hypothalamus, leptin exerts an anorexigenic power, binding to its receptor (LepR-b) and activating a signaling pathway, which induces the activation of proopiomelanocortin (POMC) neurons and the inhibition of releasing agouti-related protein (AgRP). The same anorexigenic power is exerted by insulin when it binds to its IRS receptor. In contrast, ghrelin exerts orexigenic effects, binding to the growth hormone secretagogue receptor (GHSR) and promoting hunger, activating AgRP neurons.

Hypothalamus and its nuclei, such as arcuate nucleus (ARC), ventromedial (VMH), paraventricular, and dorsomedial hypothalamus and the lateral hypothalamic area, play a fundamental role in the regulation of energy and glucose metabolism through the response to peripheral circulating signals, such as signals of a hormonal nature (e.g., leptin and insulin), as well as nutrients (glucose and fatty acids). Neurons in arcuate nucleus (ARC), such as neurons expressing anorexigenic proopiomelanocortin (POMC) and orexigenic agouti-related protein (AgRP)/neuropeptide Y (NPY), are involved in the regulation of energy and glucose homeostasis, through their response to leptin and insulin signals or their interaction with gut hormones, especially ghrelin, glucagon-like peptide-1 (GLP-1), peptide YY3-36, cholecystokinin, and pancreatic polypeptide. It is crucial to study the role of leptin and insulin at the neuronal level. In particular, it has been observed that in hypothalamus, leptin exerts an anorexigenic power, binding to its receptor (LepR-b) and activating a signaling pathway, which induces the activation of STAT3 with subsequent POMC neurons’ activation and NPY/AgRP/GABA neurons’ inhibition. The same anorexigenic power of leptin is exerted by insulin, when it binds to its IRS receptor, activating a phosphorylation cascade. The leptin receptor and insulin receptor are coupled in a common signaling pathway through phosphatidylinositide-3-kinase (PI3K) in POMC neurons. In contrast, ghrelin exerts orexigenic effects, linking to its receptor, the growth hormone secretagogue receptor (GHSR), and promoting hunger and lipid metabolism in different organs, activating AgRP neurons and inhibiting POMC neurons [18,57,58] (Figure 2).

It is worth noting that POMC neurons reduce food intake and increase energy expenditure, while AgRP/NPY neurons exert the opposite effects. These two neuronal populations play an antagonistic role in modulating glucose metabolism, even though the intracellular mechanisms involved in their activation are not fully clear.

Hypothalamic mitochondria are involved in the modulation of energy balance, modulating the neuronal activity of AgRP and POMC [34,59,60]. Excess food intake can induce mitochondrial dysfunction with consequent production of ROS and oxidative stress, as previously discussed [18,61].

Several studies on animal models, indeed, have shown that diet-induced obesity causes brain mitochondrial dysfunction indicated by an increase in mitochondrial ROS formation and an increase in the mitochondria’s size, responsible for impaired glucose metabolism. Furthermore, it appears that the antioxidant activity of enzymes, such as SOD, CAT, and GPx, is blunted, and the neuronal activity of both POMC and AgRP is driven by ROS [18,62].

Many studies, indeed, have reported structural and functional alterations of mitochondria due to the selective deletion of Mfn2 or Mfn1 in POMC neurons. In particular, this dysfunction is accompanied by abnormal glucose homeostasis due to reduced pancreatic insulin secretion. Furthermore, the response to hormonal signals, in particular the response to insulin by POMC neurons, was also compromised. In the regulation of glucose homeostasis and leptin responsiveness, proteins play a role related to mitochondrial fission such as DRP1. It has been reported, indeed, that a deletion of DRP1 in POMC neurons improves sensitivity to glucose and leptin [57,63,64,65].

It has been shown, however, that the selective deletion of Mfn1 and Mfn2 in AgRP neurons influences energy metabolism, inducing weight loss. The deletion of these proteins, indeed, appears to cause an alteration in the size and density of mitochondria in AgRP neurons, compromising the activity of these neurons [66,67].

Paraventricular nucleus of hypothalamus (PVN) and its neurons play an important role in controlling stress, metabolism, growth, reproduction, immune, as well as other autonomic functions [68]. PVN neurons play an endocrine function through the secretion of oxytocin or vasopressin directly into the circulation [69]. It has been shown that the regulation of energy balance can also be influenced by the release of oxytocin. Studies have shown that rodents with diet-induced obesity (DIO) or genetically obese with impaired or defective leptin signaling treated with chronic systemic administration of oxytocin showed a reduction in food intake, suggesting a potential use of oxytocin as a therapy for counteracting leptin resistance. According to this evidence, an interruption in oxytocin signaling can cause alterations in both food intake and energy expenditure, contributing to the emergence of obese phenotypes [70]. However, further studies are needed to understand its role in regulating body weight.

Ventromedial hypothalamus (VMH) is an important brain region involved in the control and regulation of energy homeostasis and glucose. It contains glucose-sensitive neurons, which are able to respond to high glucose, as well as to low glucose levels; moreover, a subgroup of VMH neurons expresses insulin receptors. However, the intracellular mechanisms underlying the ability of VMH neurons to detect and respond to changes in peripheral glycemic levels in the control of glucose homeostasis are still unclear. Recent evidence, moreover, has suggested that mitochondria, in particular mitochondrial dynamics, play a role in these mechanisms, influencing neuronal activation and therefore systemic glucose [71]. Some studies, indeed, have shown that DRP1, the protein involved in mitochondrial fission, is regulated by uncoupling protein 2 (UCP2), a mitochondrial protein that plays a fundamental role in the detection of glucose and, sequentially, in the regulation of systemic glucose metabolism in VMH neurons [72].

## 5. Glial Cells, Microglia, and Mitochondrial Dynamics

Recent studies have shown that mitochondrial dynamics are also involved in the activation of microglia in the pathogenesis of several diseases, but the microglial activation process is still largely unknown [20,73].

Microglia cells are highly dynamic, belonging to glial cells, which constitute more than 50% of the central nervous system mass; these cells are involved in many aspects of the nervous system function, such as the formation, plasticity, and maintenance of neural circuits. Moreover, they facilitate neuronal survival and function; finally, these cells appear to be involved in body weight homeostasis and obesity.

Glial cells, according to the different subsets, based on their morphology, function, and location in the nervous system, are generally divided into two main glial subsets, macroglia, including astrocytes and oligodendrocytes, and microglia.

Macroglia cells are commonly considered tissue-supporting cells, while microglia cells are considered the “immune cells of brain”.

Microglia cells represent the first population of glial cells discovered in brain. These cells are generated simultaneously with the neuronal precursors during the early development of embryonic brain [21,74,75].

Previous studies have shown that microglia cells are derived from yolk-sac primitive myeloid progenitor cells. Yolk-sac-derived macrophages invade brain at the early embryonic stages, representing then the vast majority of microglia in the adult. This mechanism has now been demonstrated in different animal species, such as zebrafish, birds, rodents, as well as humans.

In the beginning, the only role recognized for microglia cells was, once activated, to help protect brain from damage and infection through phagocytosis and the secretion of chemokines and cytokines, in response to pathological triggers. Conversely, recent studies have demonstrated a greater role, recognizing that these cells were not “resting cells”, but highly dynamic cells. Both in physiological and pathological conditions, in addition to their role in the regulation of tissue homeostasis by exerting an immune function, they are able to influence synaptic transmission and synaptogenesis, contributing to the maturation of neural circuits. Furthermore, microglial cells are able to receive and reply to local neural activities, through the expression of specific receptors and their ability to secrete neuroactive molecules [76,77].

They are able to change shape constantly and move and examine the surrounding environment to detect alterations to induce homeostasis, with appropriate responses. Microglial cells have been suggested to be involved in the regulation of neuronal number development; furthermore, these cells can also modulate neuronal activity through the promotion of synaptic plasticity and the release of neurotrophic factors and anti-inflammatory cytokines [78,79,80,81]. In physiological conditions, the microglia cells maintain a healthy local environment by detecting anomalies or particular substances. In pathological conditions, such as inflammation induced by a diet rich in fats, microglia could be activated, synthesizing and releasing cytokines, reactive oxygen species (ROS), and nitric oxide (NO), consequently triggering pro-inflammatory responses [82,83,84,85,86].

Recent studies have shown that microglia activation plays a pivotal role in the development of hypothalamic inflammation in obesity; understanding the role played by the microglia in the pathogenesis of this disease could represent a way in the development of new therapies to prevent or counteract obesity [86].

**Figure 3:**
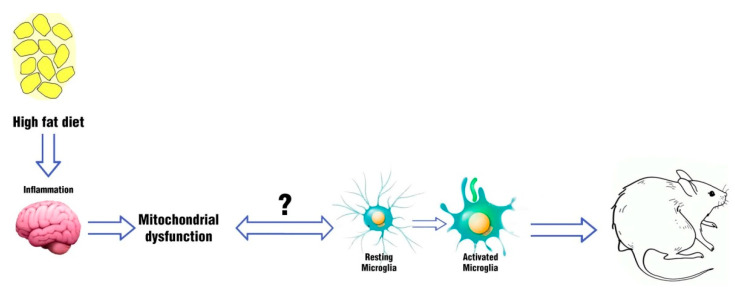
Effects of inflammation induced by a diet rich in fats on the non-neuronal cell population.

In 2012, Thaler et al. revealed that, in a rodent model with obesity induced by the consumption of a high-fat diet (HFD), hypothalamic inflammation occurred, both in rats and mice, within 1-3 days of the HFD start, before changes in body weight occurred. Furthermore, within the first week of HFD, the markers related to the neuronal lesion increased both in hypothalamic arcuate nucleus (ARC) and in the adjacent median eminence (ARC-ME) with consequent reactive gliosis, inducing the recruitment of astrocytes and the activation of the microglia. These data suggested that in a rodent model sensitive to diet-induced inflammation, the consumption of an HFD induces microglia activation and hypothalamic inflammation, causing an injury in a brain area that plays a key role in energy homeostasis. According to these results, they suggested a potential link between obesity and hypothalamic damage in animal models, as well as in humans [87]. In 2014, Valedearcos et al. showed that inflammation, as well as gliosis and neuronal stress in basic hypothalamus (mediobasal hypothalamus (MBH)) induced by a diet rich in saturated fat (SFA) could be mediated by microglia activation. Their data, indeed, have shown that in a mouse model, the inhibition of microglia was able to block inflammation and neuronal stress in ARC induced by excessive consumption of saturated fats; moreover, the inhibition of microglia activation improved the signaling of leptin and reduced the intake of food [88]. In a recent study (2017), it was demonstrated that hypothalamic and peripheral inflammation induced by diet could be prevented, inhibiting microglia expansion. In particular, the authors observed that mice under high-fat diet (HFD) showed an increase in body weight, fat mass, and in the number of microglia cells in arcuate nucleus of hypothalamus compared to standard diet-fed mice. They treated mice with the antimitotic drug arabinofuranosyl cytidine (AraC), effective at preventing body weight gain and increasing the number of activated microglial cells in arcuate nucleus. According to the previous studies, their results suggested that inhibition of diet-dependent microglia activation may represent a pharmaceutical target to prevent obesity [89]. Although the microglial activation process is not yet fully understood, the studies discussed previously showed that microglia activation could be involved in the regulation of metabolic dysfunctions. Moreover, taking into account that processes such as changes in microglia activity require energy, the role of mitochondrial dynamics in microglia activation in hypothalamus has been intensively studied in recent years, to try to build a new perspective on non-neuronal metabolic regulation, which could represent a road to new therapies to treat obesity (Figure 3). In 2017, Yi et al. demonstrated that in obese mice, induced by a hypercaloric diet, activated microglia in MBH caused TNFα secretion that stimulated mitochondrial ATP production in POMC neurons, promoting mitochondrial fusion. Their data showed that microglia activation and consequent TNFα secretion induced mitochondrial stress in POMC neurons that contributed to the development of obesity, suggesting TNFα downregulation to prevent this pathological condition [90].

In 2017, Katoh et al., in a mouse reactive microglia model, demonstrated that stimulation of TLR4 with lipopolysaccharide, already used in previous studies to evaluate microglia reactions, induced the activation of mitochondrial fission protein 1 (FIS 1) and dynamin-related protein 1 (Drp1), as well as an increase in the production of reactive oxygen species (ROS). Their results showed that microglial reactions were able to induce mitochondrial dynamics processes, such as mitochondrial fission/fusion associated with different signaling pathways; mitochondrial dysfunction appeared to contribute to the different functions of the reactive microglia involved in neurological diseases [91].

In 2018, using a male rat model, it was found that chronic HFD consumption caused a lack of hippocampal plasticity, as well as mitochondrial dysfunction and microglia activation, inducing systemic inflammation of the intestine and impaired peripheral insulin sensitivity. Rats were treated with prebiotics, probiotics, or symbiotics; this treatment was able to improve hippocampal plasticity, mitigate cerebral mitochondrial dysfunction, and reduce microglial activation involved in obesity-insulin resistance [92].

In an in vivo study (2019), it was reported that obesity induced by an HFD determined early activation of microglia and hypothalamic inflammation with hyper-expression of uncoupling protein 2 (UCP2) mRNA associated with mitochondrial dysfunction. Consequently, the pivotal role of microglia activation was suggested in the development of hypothalamic inflammation in obesity. On the contrary, selective microglial deletion of UCP2 protected mice from weight gain and hypothalamic inflammation. These results suggested a close relationship between mitochondrial dysfunction and microglia activation [22].

Mitochondrial dynamics involvement in microglial activation has been observed also in other diseases, such as neurodegenerative diseases. In previous studies on degenerative diseases, indeed, an involvement of mitochondrial fission was observed in the production of mitochondrial ROS in activated microglial cells, influencing the expression of pro-inflammatory mediators through the activation of factor nuclear kappa B (NF-κb) and mitogen-activated protein kinase (MAPK), representing a new therapeutic approach for preventing neurodegenerative diseases [73,93]. Many studies showed that hypercaloric environments stimulate microglial inflammatory activation, inducing a multicellular hypothalamic response and obesity susceptibility. These results support the view that a new non-neuronal target is on the way for treating metabolic diseases, but at the moment, further studies are required to clarify the mitochondrial dynamics involvement in microglial activation in metabolic diseases [88,94,95].

## 6. Conclusions and Future Directions

The regulation of energy and glucose homeostasis is essential for the maintenance of life. Central nervous system cell populations play a key role in regulating the energy balance, and for this purpose, they require energy; mitochondria provide a constant energy supply to these cells. Mitochondrial dynamics, in particular fusion and fission processes, are important and essential for maintaining cellular homeostasis: several studies have shown that mitochondrial dysfunction can be linked to a number of diseases, such as obesity and diabetes [18]. These mitochondrial processes, indeed, can play a pivotal role in the general metabolism of the body, in particular in the activity of the neuronal populations involved in the regulation of energy homeostasis and glucose, through mechanisms that affect the production of reactive oxygen species and inflammatory cytokines. In particular, hypothalamic mitochondria have been shown to modulate the energy balance, influencing the neuronal activity of AGRP and POMC cells. Furthermore, the changes in mitochondrial metabolism, such as in inflammation, can contribute to the activation of the microglial system in several diseases; however, further studies are needed to clarify the involvement of mitochondrial dynamics in the activation of microglia in hypothalamus. These studies are opening a new perspective on non-neuronal metabolic regulation, important for the development of new therapies to prevent and counteract obesity.

## Figures and Tables

**Table 1:** Mitochondria play an important function in energy metabolism, and a dysfunction of their processes, due to mutations in some mitochondrial genes (in particular mitofusin, Opa1, and Drp1), is implicated in the pathophysiology of obesity and diabetes.

Mitochondrial Proteins	Type of Mutation	Mitochondrial Dynamics Processes/Tissue or Cells	Obesity-Associated Disorders Related to Mitochondria Dysfunction
Mfn1	Deletion	Impaired fusion/POMC neurons	Defective insulin secretion and abnormal glucose homeostasis
Mfn2	Reducedexpression	Impaired fusion/skeletal muscle	Type 2 diabetes
	Deletion	Impaired fusion/skeletal muscle and liver	Glucose intolerance and enhanced hepatic gluconeogenesis
	Ablation	Impaired fusion/POMC neurons	Leptin resistance and decreased energy expenditure
Opa1	Deletion	Impaired fusion/pancreatic β cell	Hyperglycemia
Drp11 *	Deletion	Impaired fission/pancreatic β cell	Insulin resistance

* Further studies are required to determine the Drp1 involvement in the pathophysiology of obesity and diabetes. Mfn1 (mitofusin 1), Mfn2 (mitofusin 2), Opa1 (Optic Atrophy Protein), Drp1 (dynamin-related protein 1).
